# Adrenocortical cancer recurrence following initial transcutaneous biopsy: a rare demonstration of needle tract seeding

**DOI:** 10.1530/EO-21-0031

**Published:** 2021-11-04

**Authors:** Nada Younes, Isabelle Bourdeau, Harold Olney, Paul Perrotte, Odile Prosmanne, Mathieu Latour, David Roberge, André Lacroix

**Affiliations:** 1 Division of Endocrinology, and Research Center, Centre hospitalier de l’Université de Montréal (CHUM), Montréal, Québec, Canada; 2 Division of Hematology and Medical Oncology, Department of Medicine, and Research Center, Centre hospitalier de l’Université de Montréal (CHUM), Montréal, Québec, Canada; 3 Division of Urology, Department of Surgery, and Research Center, Centre hospitalier de l’Université de Montréal (CHUM), Montréal, Québec, Canada; 4 Department of Radiology, and Research Center, Centre hospitalier de l’Université de Montréal (CHUM), Montréal, Québec, Canada; 5 Department of Pathology, and Research Center, Centre hospitalier de l’Université de Montréal (CHUM), Montréal, Québec, Canada; 6 Division of Radiation Oncology, and Research Center, Centre hospitalier de l’Université de Montréal (CHUM), Montréal, Québec, Canada

**Keywords:** adrenocortical carcinoma, needle biopsy, metastasis

## Abstract

**Summary:**

Needle tract seeding is a potential, albeit rare, complication following transcutaneous biopsies, leading to the seeding of tumor cells along the path of the needle. Biopsies of adrenal masses are not routinely recommended and are only indicated, after exclusion of pheochromocytoma, when an adrenal metastasis of a primary extra-adrenal cancer is suspected or when pathological confirmation of inoperable adrenocortical cancer (ACC) may impact treatment. Despite guideline recommendations to avoid primary adrenal biopsy, very few needle tract seeding cases have been reported and none were in the context of an ACC. We report the occurrence of needle tract seeding in a patient following adrenal transcutaneous biopsy leading to ACC abdominal wall recurrence.

**Learning points:**

- Needle tract seeding is a rare complication of transcutaneous biopsy. It may increase morbidity and impact overall survival. It has yet to be documented in adrenocortical carcinoma (ACC).
- Adrenal masses can be accurately evaluated for malignancy using a combination of conventional and metabolic imaging, such as CT and fluorodeoxyglucose-PET, obviating the need for biopsies.
- Adrenal mass biopsy is not indicated in ACC unless advanced ACC is diagnosed, and a pathological confirmation would impact management.

## Background

Tumor seeding or needle tract implantation is a rare complication of percutaneous biopsy in which malignant cells are implanted along the trajectory of the needle, thereby increasing cancer recurrence risk (Robertson & Baxter 2011). The estimated risk of needle tract seeding largely depends on the site of the tumor to be biopsied. It was shown to be around 0.061% for pulmonary nodules, up to 4% for pleural malignancies, 0.13–2.7% in hepatocellular carcinoma, less than 0.01% in renal biopsies and 0.009% overall in abdominal biopsies (Robertson & Baxter 2011). Other factors, in particular, large tumor size, poorly differentiated and high-grade tumors, superficial abdominal masses, large needle diameter and withdrawal of needle without negative pressure may play a role in increasing the risk of seeding while the number of needle passes may not (Robertson & Baxter 2011, Tyagi & Dey 2014). To date, no estimated risk is determined for adrenal biopsies, and only a few cases of tumor seeding following percutaneous biopsy of adrenal masses have been described in the literature, namely in the context of metastatic lung carcinoma (Habscheid *et al.* 1990, Voravud *et al.* 1992). Needle tract-related metastases or recurrences can be detected up to 4 years following initial biopsy but may occur earlier in aggressive tumors (Tyagi & Dey 2014). According to the European Society of Endocrinology Clinical Practice Guidelines on the management of ACC in adults (Fassnacht *et al.* 2018), a routine biopsy of adrenal mass suspected to be malignant is not recommended, unless a metastasis from extra-adrenal malignancy is suspected or the patient is inoperable and a histopathologic diagnosis is required. We describe a unique case of ACC recurrence secondary to needle tract implantation of malignant adrenocortical cells 2 years after initial biopsy despite adjuvant therapy with mitotane at therapeutic doses.

## Case presentation

A 43-year-old woman with no significant previous medical history noted progressive abdominal fullness and left flank pain. Her family history was significant for breast cancer in her mother and maternal grandmother at ages 60 and 45, respectively. An abdominal ultrasound was remarkable for a left flank mass presumed to be secondary to splenomegaly. A lymphoproliferative syndrome was thus suspected, and a fluorodeoxyglucose-PET (FDG-PET) scan was ordered and identified a single left 15-cm retroperitoneal abdominal mass, displacing the stomach, pancreas and anterior cortex of the left kidney. The mass was highly avid on FDG-PET (standardized uptake value, SUV_max_ 22) with area of calcification and necrosis. No other hypermetabolic lesions or adenopathies were found. The consultant hematology–oncologist at a regional hospital requested a transabdominal biopsy of the mass, which was performed in June 2017, with an 18-gauge needle from an anterior approach without image capture of the procedure. Biochemical exclusion of pheochromocytoma was not performed prior to the biopsy of the abdominal mass as it was believed to be of splenic origin. Following histological examination, a diagnosis of ACC was suspected by the local pathologist who requested consultation from the adrenal pathology expert at our institution, who confirmed the probable diagnosis of ACC.

## Investigation

The patient was referred to our quaternary adrenal tumor center. An extensive pre-operative endocrine evaluation determined the tumor to be secreting adrenal androgens and cortisol at low levels without clinical signs of hyperandrogenism and hypercortisolism (Table 1).

**Table 1:** Laboratory hormonal evaluation at diagnosis of ACC, June 2017.

Hormone	Patient level	Normal levels
Plasma metanephrines	<0.17 nmol/L	<0.48 nmol/L
Plasma normetanephrines	0.22 nmol/L	<1.08 nmol/L
Morning plasma cortisol^a^	73 nmol/L^b^	<50 nmol/L
Plasma cortisol PM	334 nmol/L^b^	60–325 nmol/L
24 h urinary free cortisol	690.9 nmol/day	153–789 nmol/day
ACTH	4 pmol/L	2–11 pmol/L
DHEAS	17 µmol/L^b^	0.95–11.67 µmol/L
Estradiol	128 pmol/L	<306 pmol/L
Total testosterone	2 nmol/L	<2.77 nmol/L
SHBG	35 nmol/L	18–114 nmol/L
Androstenedione	15.9 nmol/L^b^	<10.9 nmol/L

^a^Following 1 mg dexamethasone at bedtime; ^b^Abnormal result.

ACTH, adrenocorticotropin hormone; SHBG, sex hormone-binding globulin.

**Figure 1:**
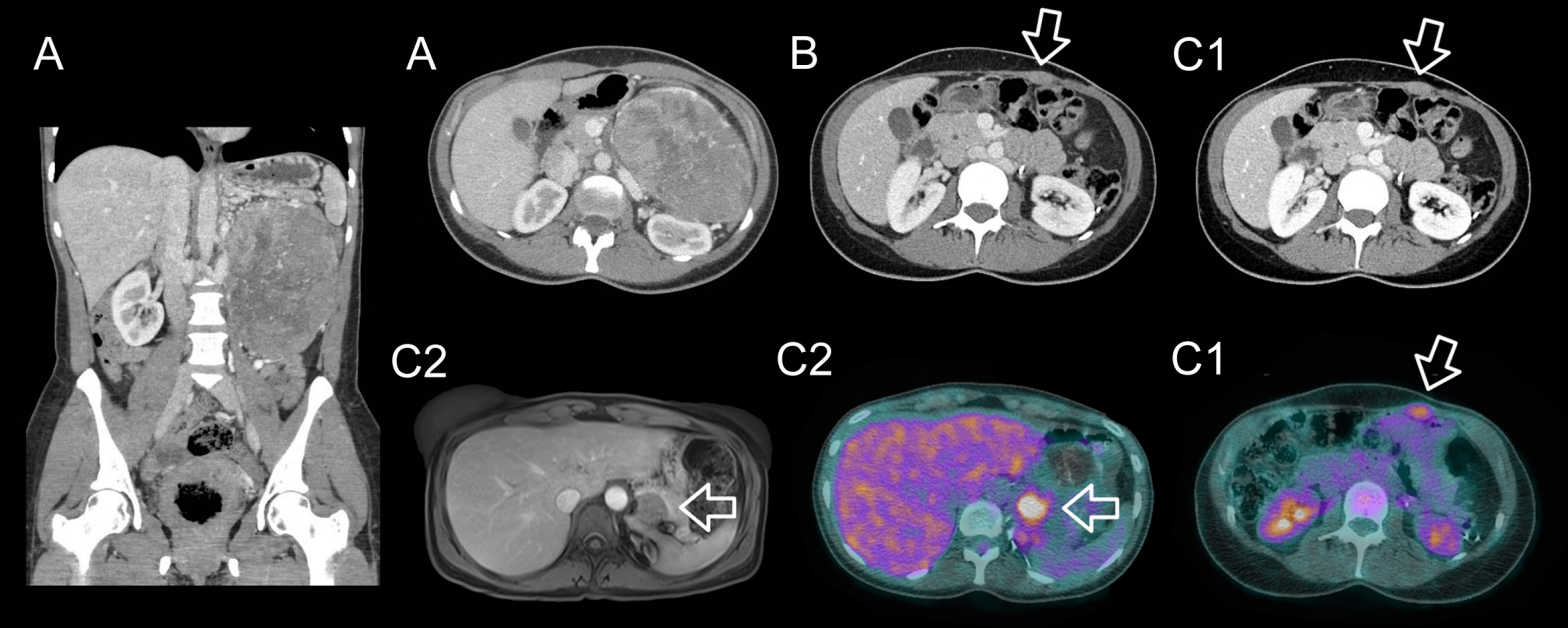
(A) A 15 cm, heterogenous, retroperitoneal mass is shown on coronal (left panel) and axial (right panel) abdominal CT imaging with i.v. contrast. (B) The first abdominal wall recurrence, 2 years following initial surgery: a 3 cm mass is seen in the left anterior abdominal wall on CT (arrow). (C) Second recurrence, 3 years after surgery. (C1) A 2.1 × 1.3 cm left anterior abdominal wall lesion is seen respectively on CT (upper panel) and FDG-PET (lower panel) (arrow). (C2) A 2.2 × 1.5 cm retroperitoneal recurrence is shown, on the superior pole of the left kidney, behind the splenic vein at the initial tumor bed, respectively on MRI (left panel) and FDG-PET (right panel) (arrow).

Thoracic and abdominal CT with i.v. contrast identified the left, vascularized, heterogenous, retroperitoneal mass measuring 15 × 11.5 × 11.4 cm, with calcifications but no regional or distant metastases (Fig. 1A).

## Treatment

One month later, open left adrenalectomy was performed at our institution. Post-operative morning plasma cortisol was very low (36 nmol/L), and the patient was discharged on 30 mg of hydrocortisone per day, in three divided doses.

**Figure 2:**
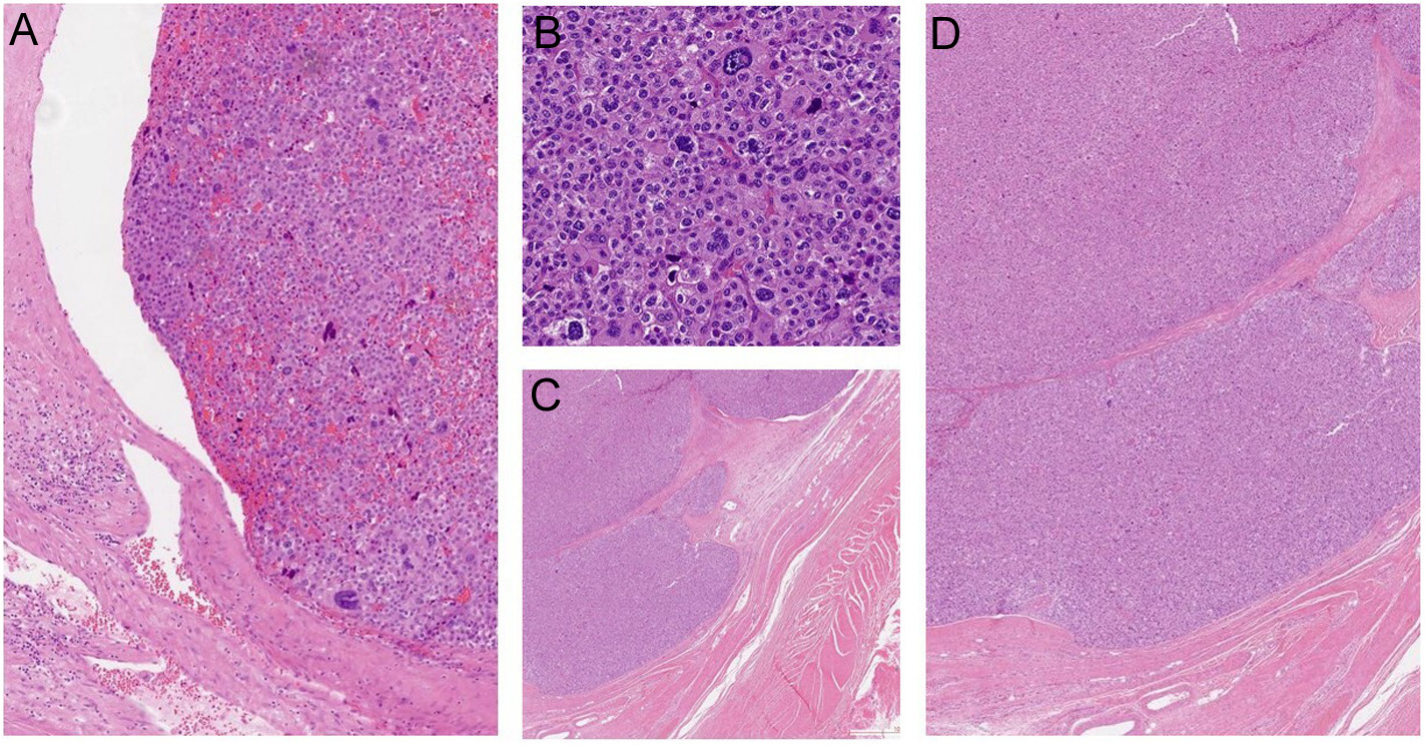
(A) Obvious vascular invasion at the periphery of the primary cortico-adrenal tumor. (B) Higher power showing marked nuclear pleomorphism and mitotic activity in primary tumor. (C and D) Recurrent tumor nodules invading the abdominal wall, showing similar morphology with the primary tumor.

Pathology was that of a high-grade ACC with oncocytic features, measuring 16 × 13 × 10 cm, a Weiss/Aubert score of 6/7, a proliferation index (Ki67) of 12%, overexpression of p53 protein, a complete surgical resection (R0) and no positive adenopathy (Fig. 2A and B).

The patient consented to medical genetic evaluation, which revealed the absence of germline mutation in current panel of oncogenic predisposition genes (*APC, ATM, BMPR1A, BRCA1, BRCA2, BRIP1, CDH1, CHEK2, EPCAM* (deletion and duplication testing only), *MEN1, MLH1, MSH2/6, MUTYH, RAD51D, NBN, PALB2, PMS2, PTEN, STR11, SMAD4 and TP53)*.

Following discussion at our adrenal multidisciplinary tumor board, adjuvant mitotane therapy was initiated at 500 mg BID and then, progressively increased to 2500 mg BID over a course of 6 months, with adequate hydrocortisone supplementation of 40 mg/day, divided into three doses. Mitotane blood levels were successfully maintained in the therapeutic range (14–20 μg/mL) at 19.7 μg/mL and one episode of toxicity up to 31.6 μg/mL, requiring reduction of mitotane to a sequential regimen consisting of 500 mg on day 1, 1 g on day 2 and 1.5 g on day 3 for a few months, followed by alternating between 1.5 and 2 g/day. Mitotane levels were consistently between 14 and 20 μg/mL on the latter regimen.

## Outcome and follow-up

Regular abdominal and thoracic imaging were performed at 3 months interval following surgery, alternating conventional CT and FDG-PET, and were initially negative for recurrences or metastases.

Two years later, the patient presented with a 3-cm mass located in the left anterior abdominal wall, which was not seen on FDG-PET scan done 3 months earlier (Fig. 1B). Biopsy of the lesion was performed using an ultrasound-guided anterior approach with an 18-gauge needle, allowing the diagnosis of abdominal wall recurrence of her ACC, presumably secondary to needle tract seeding during transcutaneous biopsy of initial mass at the time of diagnosis. The recurrence was surgically resected and documented on histopathology as a recurrence of the poorly differentiated ACC (Fig. 2C). The patient declined adjuvant radiotherapy at that time, and adjuvant treatment with mitotane was maintained (blood levels between 18 and 22.2 μg/mL) despite the recurrence.

**Figure 3:**
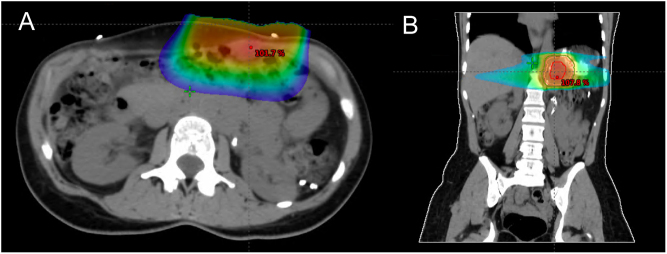
Axial (A) and coronal (B) CT imaging of radiation therapy planning targeting the anterior abdominal wall recurrence (A) and the retroperitoneal mass at the initial tumor bed (B).

One year later, she developed two new recurrences (Fig. 1C1 and C2): a 2.2 × 1.5 cm lesion located on the superior pole of the left kidney, behind the splenic vein at the initial tumor bed and a 2.1 × 1.3 cm abdominal wall lesion, both highly avid on FDG-PET, SUV_max_ 8.5 and 4.5, respectively. Following CHUM adrenal tumor multidisciplinary team’s discussion noting the rapid extended recurrence, we decided to first administer 2500 cGy in five fractions to each of the abdominal wall and the left adrenal bed recurrences (Fig. 3), followed by surgical resection 1 month later; unfortunately, minimal tumor rupture occurred during pancreas dissection. The pathology study showed an abdominal wall high-grade ACC with oncocytic features, measuring 3.5 × 3 cm with free margins and lymphatic invasion and a retroperitoneal ACC with oncocytic features, measuring 2.5 × 2 × 2 cm with positive margins (Fig. 2D). An FDG-PET done 3 months later showed no signs of recurrence. Approximately 3 months post-operatively, adjuvant chemotherapy, currently under investigation in high-risk patients (NCT03583710), was administered using cisplatin 60 mg/m^2^ IV over 2 h on day 1 and etoposide 100 mg/m^2^ IV over 2 h on days 1–3 every 21 days, for a total of four cycles, the last in June 2021. Concomitantly, we maintained treatment with 1 g of mitotane daily, 27.5 mg of hydrocortisone in three divided doses and a therapeutic mitotane blood level of 15.9 μg/mL. Close follow-up imaging is planned at 3-month interval.

## Discussion

This report provides a unique demonstration of ACC recurrence in the anterior abdominal wall, following transcutaneous biopsy of adrenal mass, despite therapeutic doses of adrenolytic adjuvant therapy with mitotane. Despite the clinical guidelines recommendation to avoid adrenal mass biopsy to prevent tumor spilling during this process, previously confirmed cases of such occurrence have not been documented prior to this report. The overall risk of needle tract seeding as a complication of transcutaneous biopsies of abdominal masses is very low, and most cases occurred in pancreatic, liver and renal carcinomas (Smith 1991). Other than the site of tumor, size and aggressivity are known risk factors for possible cancer cell implantation along the needle track (Robertson & Baxter 2011, Tyagi & Dey 2014). The large tumor size, as well as the documented vascular invasion on histopathology of the initial ACC biopsied in our patient, might explain the tumor’s susceptibility for seeding. However, seeding after adrenal mass biopsies is less frequently documented, perhaps because adrenal biopsy is most often not indicated and therefore less commonly performed compared to other tumor sites and a significant number of biopsies, from 10,766 to 66,397, depending on the study, were needed in order to detect this rare form of transcutaneous biopsy complication (Smith 1991). In addition, the adrenal mass biopsy is often not indicated (Fassnacht *et al.* 2018). In a retrospective study including 75 patients diagnosed with ACC who underwent transcutaneous adrenal biopsy, although, overall survival rates were similar between biopsied patients and the control group, in stages I–III ACC, there was no improved outcome with biopsy (Williams *et al.* 2014). It did, however, expose patients to unnecessary risks – 11% of patients developed complications: most commonly bleeding, hematoma or thrombo-embolism. A single case of needle seeding of ACC liver metastases from the transhepatic approach has been described (Habscheid *et al.* 1990). Diagnostic sensitivity of transcutaneous adrenal biopsy for establishing correct ACC diagnosis is around 70% (Williams *et al.* 2014, Bancos *et al.* 2016), compared to 87% for metastases to adrenals (Bancos *et al.* 2016). Also, pathologists may miss the diagnosis of ACC, since fine-needle aspiration cannot properly differentiate between adenoma and carcinoma, as compared to core needle biopsy (Lockhart *et al.* 2002, Fassnacht *et al.* 2004). Hence, it is recommended that adrenal biopsy only be performed in cases where metastasis to the adrenal gland is suspected in a patient with a history of known extra-adrenal neoplasia (Arellano *et al.* 2003, Bancos *et al.* 2016, Fassnacht *et al.* 2018). It is also crucial to exclude pheochromocytoma prior to performing a biopsy to avoid serious hypertensive complications (Bancos *et al.* 2016). Furthermore, an adrenal mass can be better characterized on imaging including CT scan, MRI and FDG-PET scan with identification of features suspicious of malignancy. Current guidelines on the management of adrenal incidentalomas (Fassnacht *et al.* 2016) recommend initial non-contrast CT imaging of the mass to rule out malignancy: in the event tumor size is ≥4 cm or density is >10 Hounsfield units (HU), the mass is considered indeterminate and either additional imaging, repeat imaging in 6–12 months, or immediate surgery is suggested to better rule out malignancy.

Other authors suggested different cut-offs to be used. Overall, tumor size less than 3 cm (Fassnacht *et al.* 2004), a pre-contrast density of less than 10 HU on CT imaging and an adrenal-spleen ratio <0.7 on in and out of phase MRI imaging, are highly predictive for an adrenal adenoma (Lockhart *et al.* 2002). However, most indeterminate lesions on imaging will be benign on biopsy, despite not fully meeting imaging criteria for adenoma (Lockhart *et al.* 2002). Bancos *et al.* recently reported that using a higher cut-off value for pre-contrast density on CT would allow for better specificity for ACC diagnosis without compromising on sensitivity and thus suggested using 20 HU as the threshold to exclude malignancy (Bancos *et al.* 2020). Additionally, positive predictive value for ACC was highest when all three criteria were present: tumor size ≥ 4 cm, pre-contrast density >20 HU and urine steroid metabolomics consistent with a high risk of ACC (Bancos *et al.* 2020). Moreover, FDG-PET imaging has become widely available and has proven to be highly predictive in distinguishing malignant adrenal lesions from benign ones (Fassnacht *et al.* 2004, Creemers *et al.* 2016), with a sensitivity and specificity of 97 and 91%, respectively (Boland *et al.* 2011). Either an SUV cut-off > 2.68–3 for adrenal uptake (Sahdev *et al.* 2010) or adrenal-to-liver SUV ratio > 1.45 (Groussin *et al.* 2009) can be used to detect malignant lesions, with a non-negligible risk of false positives ranging from 3 to 13% (especially pheochromocytoma, infectious and inflammatory disease, atypical adenoma). Altogether, this shows that adrenal biopsy can be avoided in the diagnostic approach to ACC. Complete *en bloc* resection of localized ACC is recommended for achieving the best possible outcome, and adjuvant mitotane therapy should be introduced for all resected ACC with a high risk of recurrence: stage III, incomplete resection and ki67 > 10% (Fassnacht *et al.* 2018), such as in our patient at initial presentation.

In recurrent and advanced ACC, treatment options include complete resection of recurrences or metastases whenever possible; however, second-line treatments such as radiation therapy, radiofrequency ablation, cryotherapy and chemoembolization can all be considered depending on site of recurrences and patient preference, and should be tailored to each patient (Fassnacht *et al.* 2018). Adjuvant radiotherapy can be combined with mitotane therapy, in stages III ACC or suboptimal initial surgery (Fassnacht *et al.* 2018, 2020). A recent study including 79 patients with stage IV ACC with low tumor burden (Boileve *et al.* 2021) reported a complete response in 13% of patients when combining mitotane therapy and locoregional therapy, either surgery, interventional radiology or radiotherapy to tumor bed with improved progression-free survival.

As for adjuvant chemotherapy, cisplatin ± etoposide with mitotane has been used as first line in high-risk settings, in particular, in aggressive ACC: Ki67 > 30%, vena cava thrombus, stage IV or incomplete tumor resection (Kimpel *et al.* 2021) or advanced ACC (Fassnacht *et al.* 2018, 2020) and gemcitabine and capecitabine or streptozotocin, as second line with or without mitotane (Ardolino *et al.* 2020, Amodru *et al.* 2021). Because we lack sufficient data on the best therapeutic approach in recurrent and advanced ACC and have essentially no data in cases of needle tract seeding following biopsy, we opted for combination therapy of mitotane, locoregional treatment followed by the chemotherapy regimen currently utilized in the ADIUVO-2 Trial (NCT03583710).

In conclusion, our case highlights the importance of following expert consensus in the management of adrenal masses, in particular, ACC. As the prognosis of advanced ACC is significantly worse than that of completely resected stage I–II tumors (Fassnacht *et al.* 2020), careful consideration should be taken before considering transcutaneous adrenal biopsy. Seeding increases tumor stage, exposes patients to additional therapy and reduces both expected disease-free and overall survival.

## Declaration of interest

The authors declare that there is no conflict of interest that could be perceived as prejudicing the impartiality of this case report.

## Funding

This work did not receive any specific grant from any funding agency in the public, commercial or not-for-profit sector.

## Patient consent

Written informed consent for publication of their clinical details and clinical images was obtained from the patient.

## Author contribution statement

N Y wrote the first draft of the manuscript. I B, H O, P P, O P, M L, D R and A L, each wrote parts of the final manuscript. All authors read and approved the final manuscript.
